# Senior Caribbean women: migration, resilience within the context of intersectionality

**DOI:** 10.3389/fsoc.2026.1409737

**Published:** 2026-03-11

**Authors:** Val Sylvester

**Affiliations:** Birmingham City University, Birmingham, United Kingdom

**Keywords:** communities, Black women, cultural diaspora, culture, first-generation senior Caribbean women, health and wellbeing, intersectionality theory, migrants

## Abstract

To date, contemporary research on intersectionality has provided limited evidence regarding how the health and wellbeing of first-generation, senior Caribbean women have been influenced by their experiences of migration following World War II to the United Kingdom (UK) and the United States (US). The push and pull factors associated with globalization and migration prompted both countries to rebuild their depleted workforces after the war through targeted recruitment strategies that included women from the Caribbean. This article is inspired by the original narratives of the senior Caribbean women who participated in my PhD study. It examines a unique aspect of Caribbean women’s experiences, focusing particularly on their memories from their late teenage years to early twenties. The article highlights the emotional challenges they faced when migrating to a new country. The article emphasizes the vulnerabilities of young female migrants as they adapt to their roles as both workers and mothers in their new environment. Additionally, it discusses the consequences of feeling unwelcome, unsupported, undervalued, and encountering social injustices. The experiences of senior Caribbean women illustrate that resilience is not an innate quality; instead, it involves navigating emotional and physical losses, economic hardships, and discrimination related to the intersections of race, gender, class and power.

## Introduction

“It is important to remember that as human beings, we are complex and unique individuals and always more than our suffering” (Angelou, 1994).

This article examines the post–World War II migration of Caribbean women to the UK and the US, focusing on how they navigated discrimination and cultivated resilience in unfamiliar and often hostile environments. Drawing on qualitative narratives from 22 first-generation senior Caribbean women who participated in my PhD research (Sylvester, 2021), the article focuses on the emotional dimensions of their lived experiences, how they interpreted the discrimination they encountered, the coping strategies they developed, and the ways these experiences shaped their emotional wellbeing.

While the original PhD study centered on the social policy barriers these women faced, limited childcare, discriminatory housing practices, restricted welfare access, and exploitative labor conditions, this article adopts a distinct analytical lens. By reinterpreting their narratives through the concepts of resilience (Ungar, 2012) and emotional labor, it highlights the intersecting forms of discrimination that shaped their daily lives and the strengths often overlooked in policy-focused analyses.

The women came from diverse Caribbean islands and socioeconomic backgrounds. Some were trained professionals seeking advancement, while others were unskilled or semi-skilled workers from economically disadvantaged communities. Their migration, spanning from before 1948 to the late 1970s, was driven by economic necessity and the promise of opportunity abroad. Many viewed the UK as the “Promised Land” and the US as the “Land of Opportunity,” only to encounter widespread racism, social exclusion, and structural inequalities that profoundly shaped their settlement experiences (Lloyd-Evans and Potter, 2002).

The demographic profile of these young Caribbean women, typically in their late teens or early twenties, including a significant number of young mothers, reveals a cohort undertaking demanding forms of labor in domestic service, nursing, caregiving, and factory work (Bryan et al., 1985). Despite the essential nature of their contributions to post-war labor markets, their experiences remain marginalized within mainstream migration scholarship (Andall, 2003; Kofman et al., 2000). Many assumed the role of primary earners within matriarchal family structures, reflecting long-standing Caribbean social norms, and their high participation in the formal economy underscored their centrality to national reconstruction efforts (National Statistics, 2003). Yet the emotional labor, resilience, and community-building practices that underpinned their survival and social mobility have been consistently overlooked in academic and policy discourse (Reynolds, 2005).

To deepen this analysis and recognize the need for a more comprehensive analytical lens, the article applies Intersectionality Theory (Crenshaw, 1989), Black Feminist Theory (Hill Collins, 1990), Critical Race Theory (Ladson-Billings, 1998), and Push–Pull Migration Theory (Lee, 1966). These frameworks illuminate how race, gender, class, generation, and historical context intersect to shape the women’s identities, opportunities, and emotional worlds (Berger and Guidroz, 2009; Yuval-Davis, 2011a). By centering their voices, the article offers a deeper understanding of the resilience that emerged from navigating structural inequalities.

### Background literature

Historically, first-generation Caribbean women have significantly contributed to the feminization of voluntary migration to the United States and the United Kingdom, helping achieve a notable gender balance. Unlike earlier trends, in which women primarily migrated for marriage or family reunification, these women often chose to migrate independently (United Nations, 2006). The term “feminization of migration” is gendered because it highlights women’s independence as the sole income earners in their families (Castles and Miller, 2004). Caribbean women are diverse, comprising varied national origins, languages, religions, and social classes. This diversity is often overlooked, especially concerning the intersections of race, gender, social class, power, and migration status. Some came from poverty-stricken communities, were young, courageous, and determined to succeed, actively seeking employment opportunities abroad in the UK and the US (Faist and Kivisto, 2010; Foner, 2009; Peach, 1998).

### United Kingdom perspective

In 1945, the United Kingdom needed labor to rebuild the country, which was war-torn and in the midst of a massive economic recession. The 1948 British Nationality Act granted British subjects who were citizens of the United Kingdom and the Commonwealth the right to enter, live, and work. Migrants were actively recruited from Commonwealth territories, such as Ireland, India, Eastern Europe, and the Caribbean. This period marked a significant impact of the Caribbean’s primary voluntary migration flow to the United Kingdom in 1948, resulting in approximately 300,000 Caribbean migrants, which continued until 1974 (Peach, 1991). According to critical sociological literature, including Gilroy’s (1992) work, the momentous migration wave that began in 1948, following World War II, marked a significant period. Many migrants holding British passports, because their countries of origin were part of the British Commonwealth, chose to migrate to the United Kingdom. These newcomers primarily originated from three key regions: the British Caribbean, the Indian subcontinent, and various nations in sub-Saharan Africa (Peach, 1991).

The UK was a popular destination for many people from the Caribbean because they shared a common language, English, and were familiar with British culture. Many first-generation Caribbean women found that upon arrival in the UK, they encountered racial discrimination and entrenched colonial attitudes that negatively affected their identities and life opportunities. For example, the demagogic politician Enoch Powell sought to incite fears of a racial war in which the nation would witness “rivers of blood” as it was overrun by a black population (Gilroy, 1992; Kibria et al., 2014). Throughout this pivotal timeframe, many Caribbean women were recruited to the UK to fill labor gaps in essential public services, including the NHS, public transportation, utilities, and manufacturing. However, these jobs were often undesirable, insecure, and offered limited opportunities for advancement. From an intersectional perspective, Caribbean women represented a diverse, heterogeneous group. While some arrived without qualifications, seeking employment, others held professional credentials such as teaching and nursing qualifications. Despite this, many faced discrimination as their qualifications were often unrecognized by UK employers, raising significant concerns about the challenges they encountered (Bhavani, 1994; Bryan et al., 1985; Tichenor, 2002).

### United States perspective

First-generation Caribbean women’s history forms part of the migration movement from the Caribbean to the United States (Kibria et al., 2014). The Immigration and Nationality Act, commonly referred to as the Hart-Celler Act, was enacted in 1965 (Immigration and Nationality Act, 1965). This legislation was significantly influenced by the Civil Rights Movement, which sought to confront and reform the prevailing racial hierarchies (Kibria et al., 2014). The Hart-Celler Act of 1965 (Immigration and Nationality Act, 1965) abolished the national origins quota system, effectively ending the racist quota system established by the 1924 legislation that had governed immigration to the United States (Thomas, 2012; Tichenor, 2002).

The Immigration and Nationality Act of 1965, also known as the Hart-Celler Act (Immigration and Nationality Act, 1965), established a preference system to guide the allocation of visas and permanent residence based on two fundamental principles. The primary principle was family reunification, established in 1965 and remaining a key feature of the United States immigration system. The other principle was that migrants with relevant occupational skills and qualifications, which were in short supply and highly sought after in the United States, were deemed desirable applicants and could apply to migrate to the Country. Influenced by these conditions, the Hart-Celler Act was enacted to address labor shortages across various economic sectors, including those requiring skilled workers, such as qualified Nurses. This led to a significant increase in the number of Caribbean migrants, especially women, seeking better opportunities in the United States. Notably, the United States’ geographical proximity and strong post-war economy made it an attractive destination for first-generation Caribbean women. Furthermore, family reunification significantly influenced this migration trend, enabling many women to enter the United States through established family-reunification channels (Bhabha, 2009). Moreover, since 1967, the flow of voluntary migration from the Caribbean to the US has seen more women than men making the journey, all in pursuit of job opportunities. From a gender-specific perspective, many of these women found it easier to migrate to the US because they met the socio-economic requirements for labor certifications, given the strong demand for domestic labor and qualified nurses (Foner, 2009).

### Theoretical perspective

Migration is a long-standing phenomenon that has shaped human societies, as people have persistently moved across the globe in search of better job opportunities and improved lifestyles. With the rise of globalization, migration patterns have changed significantly, offering new opportunities that are transforming the reasons and ways individuals relocate. This section then provides a brief overview of global migration theories relevant to the discussion on voluntary migration waves. Here, the focus is on Caribbean women and how their migration experiences, shaped by the intersections of gender, race, social class, and power, influence their decision to migrate (Castles and Miller, 2004).

### Migration theories

The seminal work in migration theory is often credited to Ravenstein (1885), who formulated sociological theories to explain the reasons behind migration and its effects on receiving societies.

Global migration has increased since World War II and has become a significant political issue.

The following seven key migration theories describe this migration wave:

- Classic Migration theory: Countries like Canada, the U. S. and Australia encourage immigration and offer pathways to citizenship, but impose restrictions and quotas (Ravenstein, 1885, 1889).
- Colonial Model: Nations such as the U. K. favor migrants from former colonies, which is evident in the post-war influx from Commonwealth countries like India and the Caribbean (Wallerstein, 2021).
- Guest Worker Model: Countries such as Germany, Switzerland, and Belgium permit temporary migration to address labor market needs, but these workers do not acquire citizenship rights after long-term residence (Martin et al., 2006).
- Illegal Migration: There is a rising trend of individuals entering countries without authorization (Faist and Kivisto, 2010).
- Neoclassical Economics and Migration: This specific theory explains migration due to labor shortages in one place and the attraction to a destination characterized by higher wages (Castles and Miller, 2004; de Haas, 2007).
- Network Theory and the New Economics of Migration (Stark, 1991), which is known as the “new economics of migration” This specific theory is based on sets of interpersonal ties that connect migrants from their place of origin through ties of kin.
- Push to Pull Model: Lee (1966) is the chosen theory to be used in this specific article because it resonates with the discussion presented.

The Push and Pull model, as explained by Lee (1966), is suitable for this article because it provides a clear, straightforward explanation of voluntary migration. Compared with the other six migration theories, this model aligns closely with the migration patterns of Caribbean women during the post-World War II era.

### Push to pull model

The Push to Pull Model, also known as the Push-Pull theory, was developed by Everett S. Lee in 1966 to explain voluntary migration. This model identifies two sets of factors that influence a person’s decision to migrate: ‘push’ factors, which drive individuals away from their country of origin, and ‘pull’ factors, which attract them to a new destination. Push factors can include limited economic opportunities, political instability, or adverse living conditions in the home country, while pull factors often consist of better employment prospects, higher wages, and improved living standards in the destination country. By considering both push and pull elements, this model provides a clear and accessible framework for analyzing why people choose to move, and it is particularly relevant to the migration patterns of Caribbean women in the post-World War II period, as it captures the complex interplay of motivations behind voluntary migration.

Migration theories, especially those by Lee (1966), initially distinguished between voluntary and forced migration using ‘push’ and ‘pull’ factors. They have acknowledged that combining these elements influences individuals (Lee, 1966). Push factors include a lack of economic opportunity and adverse conditions in the home country, such as limited job opportunities. In various combinations, these factors contribute to the migratory push. Pull factors are job opportunities, higher wages than in the homeland, and are appealing aspects of the destination country, such as strong labor markets and better living conditions.

Criticism has arisen regarding the oversimplification of specific theories in their attempts to explain complex migration processes. As a result, scholars are increasingly focusing on global migration patterns as systems shaped by the interactions between macro-level and micro-level factors. The push-and-pull model is often overshadowed by more recent approaches, such as network theory, developed by Massey et al. (1993) and Massey and Taylor (2004).

In this article, the Push-Pull theory is applied to capture the significant numbers of voluntary migration of young Caribbean women to the United States and the United Kingdom since 1945. Castles and Miller (2004) suggest that, given the feminization of migration, more women are likely to migrate today. However, from an intersectional perspective, many of these young women had never left their Caribbean homeland, let alone set out to travel to another country. Therefore, these first-generation Caribbean women were unprepared and faced unknown challenges upon arrival in their new country.

Cohen (1997) describes women’s movement from the Caribbean to new countries as a voluntary migration from their homelands. This phenomenon clearly exemplifies cultural diaspora, a process profoundly shaped by permanent migration and by the powerful influences of literature, political ideas, religious beliefs, music, and evolving lifestyles. As individuals or groups relocate, they carry with them the essence of their culture, which then intermingles with new environments in impactful ways. Literature serves as a vessel for their stories and traditions, while political ideas inform their interactions in society. Religious beliefs forge strong community ties and moral frameworks. Music captures the depth of their experiences, and diverse lifestyles are adapted to reflect their new realities. Collectively, these elements create a vibrant and dynamic cultural landscape, showcasing the adaptability and resilience of identity in a global context (Cohen, 1997). In this context, first-generation senior Caribbean mothers play a pivotal role in teaching their children about their historical legacies and migration, as these are essential to transmitting Caribbean cultural values and identity (Reynolds, 2005).

This section emphasizes that historical feminist discourse has frequently overlooked the experiences of Black women, who have been marginalized due to a lack of recognition in dominant sociological theories of feminism. These theories often focus on differences but fail to address the diverse positions of Black women. Consequently, the voices and experiences of Black women have frequently been silenced in feminist debates (Williams, 2010; Yuval-Davis, 2011b).

To address this gap, the following section presents three specific theoretical frameworks for examining the experiences of senior Caribbean women who migrated to the United States and the United Kingdom after World War II. This discussion incorporates Intersectionality Theory (Crenshaw, 1989), Critical Race Theory (Ladson-Billings, 1998), and Black Feminist Theory (Hill Collins, 1990). By doing so, it offers a comprehensive methodological approach to analyzing their unique narratives.

## Theoretical influence: intersectionality theory, critical race theory and black feminist theory

### Intersectionality theory

Intersectionality is a compelling framework that underscores the complex ways in which various aspects of identity interact to create distinct forms of inequality. It thoroughly examines a wide array of factors, including race, class, gender, sexuality, nationality, religion, physical ability or disability, ethnicity, age, and other social systems that foster subordination and exclusion. Epistemologically, the history of what is now called intersectional thinking is long, and many point to Sojourner Truth’s famous speech during the first wave of feminism as one of the earliest demonstrations of intersectionality. Indeed, intersectional analysis, before it became ‘mainstreamed’, was carried out for many years mainly by Black women (Yuval-Davis, 2011b). This concept highlights the significant and interconnected impact that these diverse factors have on social status, identities, and positions within structures of power and privilege (Berger and Guidroz, 2009; Yuval-Davis, 2011b). Furthermore, these intersecting factors not only deepen the complexities inherent in intersectionality but also play a crucial role in the formation of overlapping identities (Crenshaw, 1989; Hill Collins, 2000).

It is vital to recognize that Caribbean women come from diverse historical and social class backgrounds, which greatly affect their experiences with social welfare. Recognizing these differences is essential for accurately representing Caribbean women in the UK and the US. The importance of intersectional analysis is evident in debates about inequality, human rights, and welfare needs, as it offers a framework for understanding the variation in Black women’s experiences.

Understanding the interconnected social inequalities of gender, race, and class is crucial for recent sociological research and for improving public services for migrants. The complex interplay of these inequalities creates a variety of social positions and identities. Brah and Phoenix (2004) highlight the significant contributions of Black feminists to intersectional analysis, a key feminist contribution to women’s studies that provides valuable insights into the diverse experiences of women and a constructive framework for assessing individual women’s lived experiences (McCall, 2005).

In this context, it is essential to acknowledge Crenshaw (1989), a Black feminist, legal scholar, and Critical Race theorist who coined the term ‘intersectionality’. Thus, intersectionality is defined as the interaction between gender, race, and other categories of difference in individual lives, social practices, institutional arrangements, cultural ideologies, and the outcomes of these interactions in terms of power and social inequality. Crenshaw developed the first theoretical framework for analyzing the intersections of gender, race, and class in the exploitation and exclusion of Black women from the labor market in the United States (Crenshaw, 1989, 1991). Around the same time, several European and postcolonial feminists, including Bryan et al. (1985), Essed (1991), James (1986), and Lutz (1991), played significant roles in developing what is commonly referred to as intersectional analysis. Notably, the writings of Bryan et al. (1985) have been influential in analyzing the experiences of Caribbean women who migrated to the UK. Therefore, an intersectional analysis is well-suited to address the unique historical contexts of Caribbean women and their subjective lived experiences of migration (Crenshaw, 1989; Hill Collins, 2009; Model, 1995). Whilst Crenshaw’s definition of intersectionality (Crenshaw, 1989, p. 139) has been developed from a US perspective, it is equally relevant to examining the narratives of Caribbean women, as it highlights the complexity of their lived experiences as marginalized individuals in post-World War II UK and the US.

Yuval-Davis (2011b) clearly attributes the current concept of intersectionality to Kimberlé Crenshaw, highlighting that the experiences and struggles of Black women have been significantly overlooked in feminist discussions regarding social policies and welfare needs (Williams, 2010). Intersectional Theory is based on the understanding that these women lead complex lives and often face overlapping identities, which can result in feelings of oppression in some areas while experiencing privilege in others. Thus, intersectionality can also be viewed as a methodology that emphasizes the interplay among race, class, gender, and power, aiming to provide more comprehensive and accurate accounts of the diverse experiences of women across different positions. This rationale supports its contribution to the analysis of Caribbean women’s narratives.

### Critical race theory

The definition of Critical Race Theory is a perspective on ethnic relations that begins from the premise that racism is embedded in legal systems and other social institutions and is a normal experience for many ethnic groups (Delgado and Stefanic, 2001). This underscores its importance because, until the creation of Critical Race Theory, race was used merely as a categorical variable rather than as a theoretical lens to explain the social conditions Caribbean women experience. Critical Race Theory recognizes that racism is endemic to American life, deeply ingrained legally, culturally, socially and psychologically (Delgado and Stefanic, 2001).

Critical Race Theory, as described by Ladson-Billings (1998), is crucial to include in the analysis because it is grounded in the distinctive experiences of Black people and challenges the taken-for-granted assumption that White people’s experiences are the norm (Ladson-Billings, 1998; Villenas and Deyhle, 1999). Critical Race theorists share two overarching tenets relevant to this article. Firstly, Critical Race Theorists argue that, given their history and experience, minority groups such as Caribbean women are uniquely able to articulate what racism means based on lived experiences. Secondly, for this reason, Critical Race Theory tends to make extensive use of narrative and biological methods to give voice to those who experience racism, making it a suitable framework for understanding how race intersects with other forms of discrimination. In this context, Critical Race Theory shares similarities with Intersectionality Theory, focusing on race and racism while also considering their intersection with other forms of subordination, such as class discrimination. Ultimately, Critical Race Theorists seek to make their own necessary contribution to advancing greater social equality. This is particularly relevant for the methodology of analyzing the narratives of Caribbean women, which reflect their migration experiences in the UK and the US (Delgado and Stefanic, 2001; Ladson-Billings, 1998; Villenas and Deyhle, 1999).

### Black feminist thought

Black feminist scholar Patricia Hill Collins (1990) introduced the concept of Black Feminist Thought to refer to the outsider-within status of Black women in sociology. Hill Collins defines key themes in Black Feminist Thought as follows: firstly, Black Feminist Thought is produced by Black women, focusing on the meaning of self-definition and self-valuation; secondly, there is the attention to the interlocking nature of race, gender and class oppression in the work of Black feminists; thirdly, Black Feminist Thought involves efforts to redefine and explain the importance of Black women’s culture (Hill Collins, 1990). Fourthly, Black women’s experiences represent a heterogeneous collective standpoint and have diverse responses to common societal challenges. Fifthly, Black Feminist Thought is closely tied to the struggles of Black women for human dignity, empowerment, and social justice (Hill Collins, 1990).

Therefore, by combining the key concepts of Intersectionality Theory, Black Feminist Thought, and Critical Race Theory, articulated by scholars such as Crenshaw (1989), Hill Collins (1990), and Ladson-Billings (1998), provides a suitable methodological framework to analyze how gender intersects with other identity factors such as class and power to shape the experiences of oppression encountered by Black women. Thus, when considered through the sociological imagination, as proposed by Mills (1959), it provides a realistic approach that enables these theories to be used as a comprehensive analytical tool to appreciate the uniqueness of each woman within the broader society (Mills, 1959).

These specific theories are therefore vital for examining the lived experiences of Caribbean women who migrated to the UK and the US after World War II. Each theory highlights racial inequalities while also addressing other forms of subordination, such as gender, class, and power discrimination. Moreover, each theory enables us to center the voices of Caribbean women rather than marginalizing them as outsiders when reflecting on their migration experiences. These theories help explain how stories, silences, and actions in everyday conversations are used by Black women to confront and resist discrimination and social injustice. Notably, from the outset, it was essential to focus specifically on how Black women utilize communication and language to make themselves visible and relevant (Crenshaw, 1989; Delgado, 1995; Hill Collins, 1990; Ladson-Billings, 1998).

Recognizing this diversity is crucial from a theoretical standpoint, as it enables analysis of the numerous factors influencing their experiences, particularly their motivations for migration. By applying the sociological imagination, this analysis offers an opportunity to explore how events that shape individual lives reflect broader social issues from various perspectives.

The sociological imagination, a concept by Mills (1959), emphasizes understanding personal troubles as public issues by contextualizing individual experiences within broader social and historical frameworks (Mills, 1959). Mills argues that this approach fosters self-awareness and personal insight, particularly in reflecting on the migration experiences of senior Caribbean women, revealing insights unavailable from other perspectives (Mills, 1959).

This insightful article delves into the unique challenges faced by senior Caribbean women, stemming from historical social injustices in accessing welfare and support. It highlights the significant impact of post-World War II migration on the health and wellbeing of these pioneering women, while showcasing their remarkable resilience and innovative strategies to overcome discrimination in their new homes (Bryan et al., 1985; Foner, 2009).

In this article, resilience is defined as the ability to recover from difficulties. Pooley and Cohen (2010) equate resilience with resourcefulness, emphasizing the capacity to effectively utilize internal and external resources to confront life’s challenges, adapt to change, and maintain a sense of control during times of adversity. This, in turn, fosters positive wellbeing (Hill Collins, 2000). This article explores the experiences of first-generation Caribbean women who migrated to the UK and the US after World War II, highlighting the challenges and discrimination they faced upon arrival. It underscores the remarkable resilience and strength these women have developed in response to their circumstances, drawing on theoretical insights from previous discussions. The following section investigates, through a qualitative research study, how Caribbean women exemplified resilience, as defined by Grant and Kinman (2013), through their unwavering determination to overcome obstacles faced during the post-war era. Furthermore, it recognizes the profound impact of these experiences on their health and emotional wellbeing (Nazroo, 2002; Torres, 1999; Wray, 2004).

The research examined the experiences of Caribbean women who migrated to the UK and the US after World War II, highlighting social inequality and injustices from discriminatory practices that limited access to welfare and social support (Sylvester, 2021).

### Reflexivity

During the qualitative research process, I remained keenly aware of my actions and responsibilities to accurately represent participants’ narratives, emphasizing authenticity and empowerment. I reflected on how to effectively integrate the three forms of reflexivity: memory, mediation, and method (Pinar, 2011). I recognized that traditional data collection methods would not provide a comprehensive understanding of the everyday realities faced by Caribbean women. My focus was on their emotions, fears, hopes, and desires, as well as on understanding how they navigated and resolved conflicts in their daily lives.

This process entailed exploring how senior Caribbean women utilized communication and language to assert their visibility and relevance, while also connecting with others who shared similar experiences. Engaging in reflexivity demanded continuous self-awareness and personal reflection as I listened respectfully to the women’s uninterrupted narratives. This approach allowed difficult memories to emerge, thus illuminating their strength and resilience.

It was crucial for me to steer clear of academic jargon and impersonal prose, as doing so could obscure the voices and lived experiences of Senior Caribbean women, rendering them unrecognizable. By utilizing the chosen theories, I created a meaningful framework that recognized the diverse identities of these women while ensuring that their voices remained central, authentic, and accurate to their lived experiences.

As a Black feminist academic and second-generation Caribbean woman, I understand the importance of reflexively examining my racial and gender identity within societal contexts. My age, social class, and identity as a Black academic may create power imbalances in this research setting. I felt honoured to be welcomed by the Senior Caribbean women who participated, and I value the unique opportunity to interview them and gather data about their lives.

Having been raised to respect my elders, I approached this research with a commitment to treating the Senior Caribbean women with dignity, while acknowledging our shared cultural norms. This approach was crucial at every stage of the qualitative research process, helping me avoid assumptions about their lives (Bryman, 2012).

In this context, power and authority were held by the participants, challenging the traditional notion of the powerful researcher. The interplay of race, class, and gender indicates that the researcher does not simply exercise power over the subject in social research. Thus, reflexivity in qualitative research involves curiosity and openness to ethically exploring the truth (Mason, 1996). This perspective enabled me to analyze data with a nuanced understanding of the lived experiences of Senior Caribbean women while considering their structural positions in both the US and the UK (Hill Collins and Bilge, 2016; Hooks, 1982; Riessman, 2008).

## Method

The original PhD study demonstrated that Narrative research (Riessman, 2008) is particularly effective in capturing the lived experiences of Senior Caribbean women who migrated to the UK and the US between 1948 and the 1970s (Sylvester, 2021). Riessman and Quinney (2005) highlight the importance of recognizing the complex meanings embedded in narratives and their contextual origins. Narratives should not be considered precise accounts of events or as fully representative of the broader world, given that their interpretation can vary significantly across different temporal and social contexts. This underscores the need to consider not only the language and meanings employed but also how lived experiences shape the narrator’s understanding.

Embracing a narrative approach cultivates trust among participants, encouraging them to share their thoughts more freely (Riessman and Quinney, 2005). It is crucial to interpret opinions within the broader cultural and structural contexts that influence these narratives. Unlike traditional structured interviews, a narrative approach allows for a more natural flow of responses and incorporates a range of perspectives, enhancing our comprehension of participants’ socio-historical contexts. It is essential to avoid interrogation techniques and minimize unnecessary interruptions (Riessman and Quinney, 2005).

The narrative approach effectively supports the study’s objectives by emphasizing elements of individual identity and collective memory (Hall, 1990; Riessman, 2008). The original PhD study process (see Sylvester, 2021) adhered to stringent ethical guidelines, including anonymizing data using pseudonyms to safeguard participants’ identities. Robust security measures were implemented to ensure proper access, storage, and disposal of information in compliance with the General Data Protection Regulation (2016), which is implemented alongside the Data Protection Act (2018). Written informed consent was secured from each participant to uphold confidentiality (Bryman, 2012).

The research methodology is founded on three key theories: Intersectionality Theory (Crenshaw, 1989), Black Feminist Thought (Hill Collins, 2009), and Critical Race Theory (Ladson-Billings, 1998). These frameworks facilitate a thorough examination of the health and wellbeing of Caribbean women within the context of migration. Utilizing a narrative methodology aligned with Black feminist principles, this study investigates how race and gender intersect with social class to shape the challenges Caribbean women face. This approach emphasizes the diverse experiences of senior Caribbean women by identifying their intersecting identities and the influence of inequality in their lives. Ultimately, these theories provide a comprehensive framework for understanding how unique histories, social classes, and cultural backgrounds impact lived experiences (Davis, 2008).

### Recruitment

The demographic patterns of Caribbean communities in the West Midlands, UK, and South Florida, US, facilitated Caribbean women’s participation in the PhD study through local, Black-led community agencies. Recruitment for the sampling group was conducted through direct interactions and phone communication with daycare centers, community projects, and Black-led churches.

### Sampling

In a qualitative study, a small, purposefully selected sample is employed (Miles and Huberman, 1994). This research utilized a purposive sampling strategy to focus on first-generation Caribbean women who migrated from the Caribbean to the UK and the US between 1948 and 1974. This method selects participants most likely to provide valuable insights by considering their unique characteristics, including age, social class, experiences, and professions (Patton, 2007).

These women share authentic experiences and valuable knowledge that enhance our understanding of the topic. The qualitative research design effectively complemented the purposive sampling strategy, allowing for a comprehensive examination of their narratives. Consequently, this approach yielded rich data that significantly contributes to our understanding of the significance and relevance of their migration experiences.

### Limitations

It is essential to note that the women were deliberately chosen to provide detailed, insightful data. However, this sample may not be statistically representative of the broader demographic of Caribbean women who migrated to the UK and the US during that time, which could limit the study’s findings.

### Data generation

At the outset of the engagement with senior Caribbean women, it was crucial to obtain written informed consent from each participant as an integral part of the research process. Narrative data were gathered through uninterrupted interviews with 11 women in the West Midlands, UK, and 11 more in South Florida, US. The study was designed to encourage naturally occurring dialogue (Edley, 2001; Patton, 2002; Riessman, 2008). Fieldwork was conducted over a 12-month period in 2015, which aligned with the original timeline for the PhD study (Sylvester, 2021).

### The interview process

In accordance with ethical reflexivity and in respect of Caribbean elders, the women chose a venue that best suited their needs for the audio-taped interviews, which typically lasted between 1 and 3 h. Each woman had complete control over the content, including where to begin, what to disclose, the order of topics, the pacing, and the level of detail. They served as the primary narrators of their own experiences. While I was an active listener, a topic guide focused on migration was provided to encourage their engagement in the research process.

In the interviews, all the women shared their journeys of migrating from the Caribbean to the UK or the US as young adults and now being retired. Their narratives reflected a shared historical experience and a profound sense of Caribbeanness, shaping their cultural identity within the wider context of the Black experience (Hall, 1990).

This sample should be understood within the context of ongoing discussions about the feminization of migration. As such, the women’s responses should be interpreted alongside broader research on migration trends in the UK and the US (Castles and Miller, 2004).

### The participants

All participants were first-generation senior Caribbean women, each representing a unique community. They resided in either inner-city or suburban areas of their host countries, primarily coming from Jamaica, St. Kitts, and Barbados. Their backgrounds reflected a diversity of histories and social classes. Below is a table listing the 22 participants, detailing their pseudonyms, employment status, marital status, motherhood status, and living arrangements.

### Analysis and interpretation of narrative data

Data analysis was an iterative process that occurred concurrently with data generation and transcription, analytic induction, and theoretical sensitivity (Green, 1998; Strauss and Corbin, 1990). This reflexive method prioritized the women’s experiences and utilized narrative analysis to amplify the voices of those who have historically been marginalized.

The decision to implement a non-interruptive process for collecting raw data was based on the suitability of narrative data analysis for this research. This approach ensured that the interpretation was both holistic and narrative-driven. Polkinghorne (1995) distinction between narrative analysis and the analysis of narratives offered a valuable framework.

The analysis aimed to illuminate the unique differences in each situation while also identifying common themes. By focusing on these themes, it sought to clarify how participants interpret their experiences (Polkinghorne, 1995) and to ensure the authenticity of Caribbean women’s stories is preserved.

Narrative analysis entails organizing data into a cohesive whole while preserving the metaphorical depth of a story. This method, referred to as ‘emplotting,’ involves arranging narrative data, actions, events, and occurrences to construct coherent stories from the analysis. The aim of this narrative approach is to understand the reasons and processes by which events transpired and how participants responded. This article underscores theories that resonate with Black women’s experiences, placing their perspectives at the center of the analysis. As the field of gender studies has evolved, there has been a growing recognition of the intersections among gender, race, and class, and of their roles in shaping social differences and power imbalances (Brah and Phoenix, 2004; Crenshaw, 1989; Hill Collins, 1990; McCall, 2005).

### Findings

Twenty-two senior women from the Caribbean participated in the initial PhD study (Sylvester, 2021) (see Table 1). The narrative analysis underscored that, as Black women, their voices are often marginalized in feminist discourse. Their distinct racial experiences, along with their diverse histories, socioeconomic backgrounds, and cultural contexts, were crucial for accurately reflecting the diversity within the Black female experience. Even among this purposive sampling group, significant diversity exists due to factors such as social class, age, power dynamics, and migration status.

**Table 1:** Participants.

Name (Pseudonym)	Employment	Qualifications	Marital status	Motherhood status	Regions	Accommodation status
Rea	Retired semi-skilled factory	No formal qualifications	Widow	Mother	UK	Rented accommodation
Etta	Retired nurse	State registered nurse	Married	Mother	UK	Homeowner
Gee	Retired semi-skilled sector	No formal qualifications	Separated	Mother	UK	Homeowner
Annie	Retired day care sector	Carer’s award	Widow	Mother	UK	Homeowner
Jade	Retired nurse	State enrolled nurse	Married	Mother	UK	Homeowner
Winnie	Retired semi-skilled sector	No formal qualifications	Widow	Mother	UK	Homeowner
Delia	Retired semi-skilled sector	Teaching assistant	Widow	Mother	UK	Homeowner
Eva	Retired professional social worker	Social work qualification	Married	Mother	UK	Homeowner
Tammi	Retired semi-skilled sector	No formal qualification	Widow	Mother	UK	Homeowner
Hennie	Retired senior nurse/Ward sister	State registered nurse/Ward sister	Widow	Mother	UK	Homeowner
Celia	Retired community nurse	State registered nurse/Midwifery	Widow	Mother	UK	Homeowner
Minnie	Retired admin worker	Secretarial certificate	Married	No children	US	Homeowner
Susu	Retired academic	Senior academic	Married	Mother	US	Homeowner
Laney	Retired Academic/Consultant	Senior academic	Married	Mother	US	Homeowner
Mimi	Retired care worker	Care certificate	Married	Mother	US	Homeowner
Lala	Retired care worker	Care certificate	Widow	Mother	US	Homeowner
Dee	Retired school teacher	Teaching award	Single	Mother	US	Homeowner
Nellie	Retired nursery school worker	Teaching award	Widow	Mother	US	Homeowner
Terri	Retired health professional	Teaching award/Senior nurse award	Widow	Mother	US	Homeowner
Pennie	Retired counsellor	Counselling award	Married	Mother	US	Homeowner
Lou	Retired professional accountant	Accountancy award	Widow	Mother	US	Homeowner
Frankie	Retired nurse	State registered nurse/Ward sister	Widow	No children	US	Homeowner

### Themes

This study examines how first-generation Caribbean senior women developed resilience in the face of challenges as young migrants in the UK and the US after World War II. Key themes include empowerment and disempowerment, as well as the intersections of race, gender, and class in their migration experiences.

Many women in this purposive sampling group emphasized motivation, coping mechanisms, determination, and self-efficacy as vital for creating a home and dealing with discrimination. Their narratives provide insights into the often-overlooked experiences of Caribbean female migrants during the post-war years.

These women demonstrated strength and resilience in their stories, often downplaying their struggles. It is important to recognize how Black women use language to assert their visibility and share their experiences of social injustice, sometimes opting for silence instead of elaboration. Analysis of their narratives revealed common themes, highlighting their vulnerability as migrant women in poor working conditions, exacerbated by a lack of support from policymakers in accessing welfare, childcare, and social housing (Williams, 2010).

**Figure 1:**
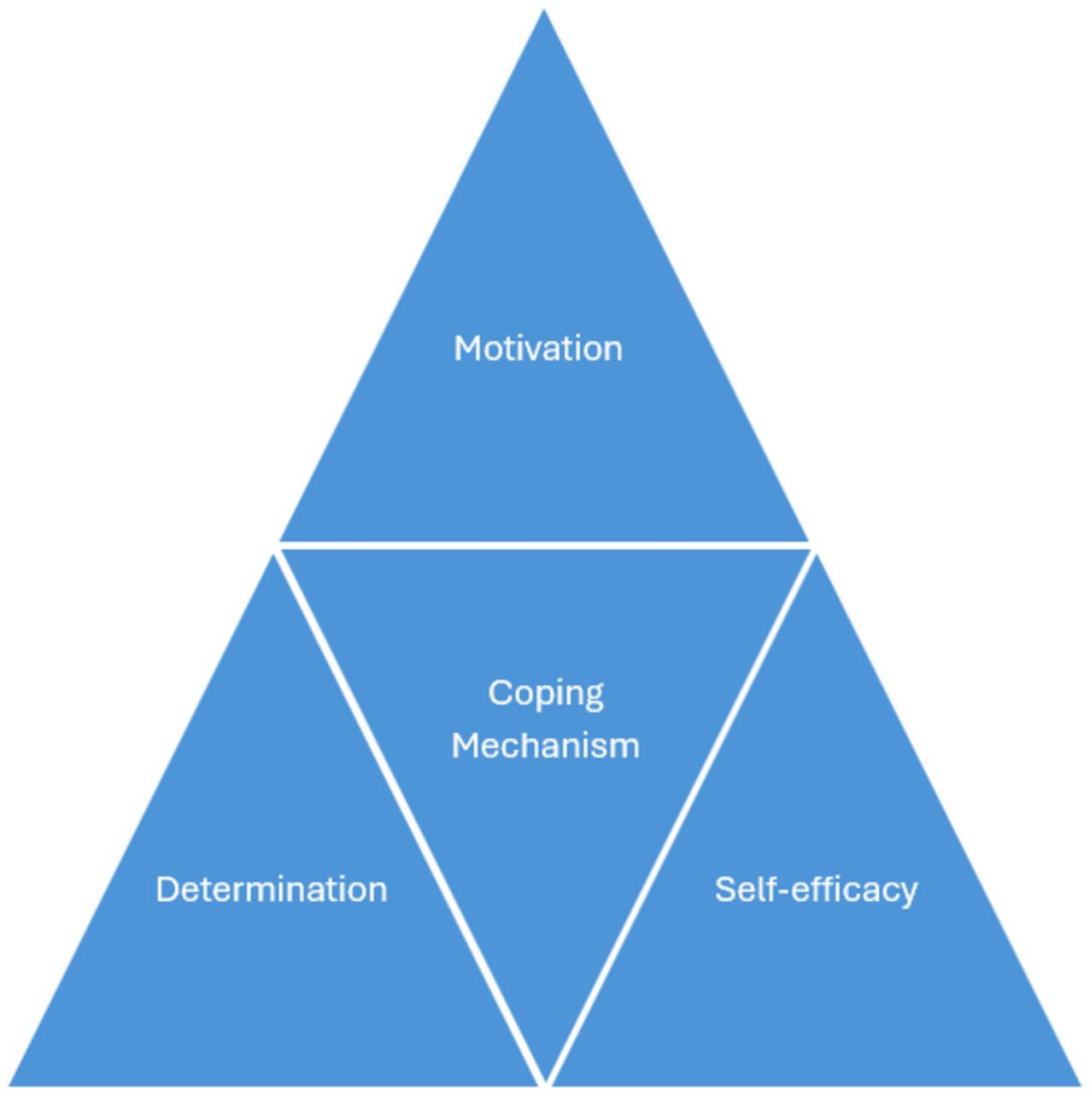
The Resilience Model (Sylvester, 2024).

The theoretical framework for interpreting and presenting the findings is a vital aspect of this article, as it provides an overview of how the key concepts were identified. It also allows for evaluating and explaining the content of the participant narratives. This paper introduces the Resilience Model, an original visual representation that illustrates four themes, deepening theoretical insight into resilience (see Figure 1) (Boylorn, 2013; Gabriel, 2004; Hill Collins, 2009; Hooks, 1982; Ibarra and Barbulescu, 2010; Barbulescu, 2010).

### The resilience model: motivation, coping mechanisms, determination and self-efficacy

Resilience is a multidimensional concept that involves various interpersonal and intrapersonal skills, playing a crucial role in the migration experiences of Caribbean women. Derived from the Latin word “resilire,” meaning “to spring back,” the term was first documented by Fonagy et al. (1994), who noted that individuals can thrive despite challenges. According to Rutter (1999) and Collin (2008), psychological resilience is the ability to maintain self-esteem in stressful situations, allowing resilient individuals to navigate life’s challenges effectively (Rutter, 1999).

Early research characterized resilience as a positive trait that helps individuals adapt to adversity. In contrast, more recent perspectives recognize it as a dynamic interplay between personal attributes and supportive environmental factors (Jacelon, 1997; Rutter, 1999). Neenan (2009) describes resilience as a flexible, cognitive, behavioral, and emotional response that enables individuals to adjust to new situations. Pooley and Cohen (2010) define it as resourcefulness, highlighting the effective utilization of both internal and external resources to maintain control in the face of challenges. Hill Collins (2008) emphasizes that managing change while remaining committed during difficult times is vital to resilience. Collectively, these definitions illuminate the strategies and strengths that many Caribbean women cultivate, often in silence, to overcome adversity in the UK and US.

In 2024, I created a Resilience Model (see Figure 1) to visually represent the narratives of Senior Caribbean women. This model, inspired by my Migration Triangle from my PhD study (Sylvester, 2021), consists of four components that highlight how these women faced adversity and demonstrated resilience (Bandura, 1977, 1986).

The Resilience Model enhances our understanding of Intersectionality, Critical Race Theory, and Black Feminist Thought, emphasizing the importance of gender in migration. It illustrates how senior Caribbean women devise strategies to overcome challenges and social injustices, demonstrating their remarkable resilience (Bandura, 1977, 1986; Crenshaw, 1989; Hill Collins and Bilge, 2016; Ladson-Billings, 1998; Lee, 1966) (see Figure 1).

This Resilience model visually encapsulates four key themes that reflect the lived experiences of senior Caribbean women. Utilising a strength-based approach, it aims to amplify their voices and counteract their marginalisation in society.

The model is constructed as a static, non-linear framework and integrates an intersectional analysis grounded in Black feminist and Critical Race perspectives. This methodology transforms women’s narratives into meaningful and impactful accounts, emphasising the roles of gender, social class, race, and culture in the contexts of migration and resilience (Crenshaw, 1989; Hill Collins and Bilge, 2016; Ladson-Billings, 1998).

The following section of the article leads into the analysis of these women’s narratives, using pseudonyms.

### Migration lived experiences

The article definitively examines personal stories through four essential categories of resilience: motivation, coping mechanisms, determination, and self-efficacy. It presents compelling narratives from participants in the UK and the US to support these findings (see Figure 1).

1. Motivation. This is a fundamental aspect of the qualitative resilience triangle and is vital for understanding the reasons behind migration from the Caribbean. It is defined as the desire to take action to pursue a goal, serving as a key driving force behind human behavior. There are two primary types of motivation: intrinsic, which originates from within an individual, and extrinsic, which is influenced by external factors.
2. Coping Mechanism. Coping is the second component of the resilience triangle, highlighting the core skills of senior Caribbean women. It is essential for accurately assessing potential threats to wellbeing.
3. Determination. Determination is a crucial component of the resilience triangle, prominently showcased in the narratives of Senior Caribbean women. This powerful quality embodies the willpower and tenacity essential for achieving success.
4. Self-efficacy. The fourth component of the resilience triangle highlights how Senior Caribbean women maintain their confidence amidst discrimination. It emphasizes the importance of achieving goals and overcoming adversity, embodying a positive humanistic quality (Duggleby et al., 2009).

#### Motivation

Migration is frequently motivated by economic factors. Pull factors, such as better job opportunities and improved living standards, entice individuals to relocate, whereas push factors, including limited job options and low quality of life, drive them to leave their home countries. For the 22 Caribbean women, migration represented an opportunity for economic advancement and a fresh start. This led them to respond favorably to recruitment invitations promising job opportunities abroad (Bryan et al., 1985; Mirza, 1997).

Lou’s motivation for migrating from the Caribbean to the US was the driving factor of employment and economic opportunity:

*I was motivated to migrate to the US because I believed it was the land of opportunity with promising employment prospects…*

[Lou, US, Accountant, mother]

Lou’s journey emphasizes the theme of motivation driven by aspiration and hope. Drawn to the United States for its opportunities, she courageously migrated in search of professional growth. Her belief in finding meaningful employment highlights her resilience and intrinsic motivation, evident even in challenging times.

The gap between expectation and reality became evident when Lou’s qualifications as a certified accountant were not recognized. She shared her experience:

*My migration from the Caribbean to the US was challenging as I struggled to find employment that aligned with my qualifications.*

[Lou, US, Accountant, mother]

Lou’s situation highlights the discrimination associated with race, gender, and power, as analyzed through a Critical Race perspective (Ladson-Billings, 1998).

Similarly, Rea moved from the Caribbean to the UK in search of better job opportunities. Although she lacked formal qualifications, she remained hopeful, yet faced numerous forms of discrimination in her quest for employment:

*I came here in 1960, and it was very cold. Finding a job was very challenging. If you went and knocked on the window, they would open it. However, as soon as they saw that it was a Black person, they would shut it down. It was hard, but we endured it because we came here.*

[Rea, UK, Factory Worker, mother].

Rea’s story is a compelling testament to her unwavering motivation in the face of rejection and hardship. Arriving in 1960 during bitter weather and colder attitudes, she faced racial discrimination that closed doors, sometimes quite literally. However, despite being shut out simply because of the color of her skin, Rea pressed on:

*Eventually, I got a job at the Jewellery Quarter, where I worked on assembly tasks. I worked there from 8 to 6, with a 1-hour dinner break. My wage was 4 pounds and 6 shillings, but we did it because there was nothing else. We had to do it to survive, as it was not easy to get a good job.*

[Rea, UK, Factory Worker, mother].

Rea’s determination to make her migration meaningful pushed her forward, even as she endured long hours in the Jewellery Quarter. Despite modest pay and exhausting conditions, she persevered, driven by a deep desire to survive and establish her place in an unwelcoming world. Working from 8 a.m. to 6 p.m. and seizing opportunities, Rea demonstrated remarkable resilience, courage, and an unwavering belief in moving forward.

Both excerpts illuminate the migration experiences of Caribbean women in the post-World War II era. Lou secured professional positions that aligned with her qualifications; however, she encountered discrimination along the way. Conversely, Rea faced racial bias that confined her to semi-skilled jobs due to her limited education. These narratives underscore the challenges many Black women experience at the intersection of race, gender, social class, and migration, often resulting in feelings of invisibility and obstructing their social mobility.

#### Coping Mechanisms

According to Mak et al. (2020), an individual’s capacity to cope is intricately linked to their perception of risk and confidence in their coping abilities. Coping mechanisms are strategies people use to navigate challenges, and these approaches can vary widely among individuals. In Gee’s narrative, she shares her experiences of migrating to the UK as a young wife facing a failed marriage. Juggling motherhood and full-time work, she persevered without family support or welfare assistance.

*I arrived in the UK at the age of 20 without any family support, except for my husband. Unfortunately, the marriage failed, and we went our separate ways, leaving me with the children…I fought along through life, sending the children to school. Working all the time. I worked three jobs to sort of keep us going. I would never encourage anyone to leave their homeland without knowing how things will work.*

[Gee, UK, Semi-skilled Worker, mother, divorced]

Gee’s narrative underscores the significance of self-empowerment in fostering effective coping strategies and resilience. This empowerment enabled her to manage her responsibilities as a full-time working single parent effectively. To ensure her children’s financial stability, she took on three jobs, reflecting her unwavering determination. By employing a problem-focused approach, she tackled challenges head-on, securing employment and prioritizing her children’s education. Ultimately, her journey vividly illustrates the themes of survival and personal agency.

Tammi’s narrative in the excerpt below demonstrates her disappointment on arrival in the UK. It is also evident that her coping mechanism evolved from an emotional and personal level, as well as the support she received from her local Caribbean community, and the story underscores her difficulty in finding a church after relocating from the Caribbean. Confronted with exclusion from traditional worship spaces, she established a supportive network with other young Caribbean mothers to create an inclusive environment. This experience highlights the challenges faced by Black women migrants, who frequently encounter discrimination and exclusion:

*You thought it would be nice to come to England, but it was terrible… When you wanted to go to the churches, they did not want to see many Black people with children attending. So, we went and found our own church. We survived because we were closer.*

[Tammi, UK, Semi-skilled Worker, mother]

Tammi’s religion and spirituality were vital sources of support for many first-generation Caribbean women who migrated to the UK during the 1950s and 1960s. Anticipating a warm welcome, they often brought letters of introduction from their church elders. While some congregations embraced them, many historic churches were either indifferent or hostile, prompting these women to establish their own, Black-led churches, including Pentecostal and Seventh-day Adventist congregations, which maintained connections with similar institutions in the US and the Caribbean (Howard, 1987; Kalilombe, 1998).

Since World War II, Black-led churches in both the UK and the US have experienced significant growth, with Caribbean women increasingly taking on leadership roles. These churches foster cultural networks and provide essential social and emotional support, empowering their members (Kalilombe, 1998).

#### Determination

Determination is the unwavering drive to achieve specific goals, enabling individuals to persevere in the face of challenges (Cambridge Dictionary, 1995). Caribbean women exemplify this quality, demonstrating a steadfast commitment to advancing their social status while embracing cultural pride, despite significant obstacles.

Nellie’s story highlights her struggles as a qualified accountant seeking to migrate to the U.S. to pursue better job opportunities. Faced with the Hart-Celler Act of 1965’s (Immigration and Nationality Act, 1965) residency requirements, she felt compelled to accept a position that did not align with her qualifications, driven by the pressing need for employment to establish residency. Unfortunately, she encountered exploitation and discrimination from her White female employer, who initially hired her as a temporary domestic worker but later refused to pay her wages.

*I had to do housekeeping because I couldn't find any other job. Once, I went to a White lady’s house to clean it. When I was done, I told her I had finished. She replied, ‘Are you supposed to be working? You'd better get out of here before I call immigration on you.’ Though I was breaking the law, honest work should not be a problem. I had to leave her place in a hurry.*

[Nellie, US, Retired Nursery School Worker, widow].

Nellie’s story highlights her resilience in confronting marginalization and job insecurity. Despite limited opportunities, she embraced housekeeping to support herself with dignity and integrity. Her willingness to take on challenging, undervalued roles underscores her strong determination to persevere despite legal and social barriers.

When faced with hostility, Nellie reacted not with fear, but with a steadfast belief that no one should be shamed for honest work. This moment underscores that dignity derives from resilience rather than recognition. Nellie’s journey illustrates the challenges faced by Caribbean women who immigrated to the U.S. in the post-war era in search of better opportunities. Without family support, they encountered significant obstacles in meeting the requirements of the Hart-Celler Act of 1965 and obtaining H-1B work visas. As a result, many were confined to domestic jobs and were particularly vulnerable to exploitation (Kibria et al., 2014).

In the excerpt below, Hennie shares her experience of successfully utilizing her Nursing Award, obtained in the Caribbean, to secure a nursing role in the National Health Service. However, after getting married, she had to leave her nursing residency and search for accommodations in the private rental sector:

*Having secured a nursing position, I married and, according to hospital policy, had to leave the hospital to live in rented, overcrowded accommodation. It was common to see signs on doors reading 'no blacks, no Irish, no dogs'. Living in one room, sharing a bathroom, kitchen, and toilet with strangers, cooking, eating and sleeping in the same space was tough... Despite these challenges, we came to the UK, so I was determined to succeed.*

[Hennie, UK, Senior Nurse, mother, married]

Hennie’s story exemplifies her quiet determination in the face of institutional barriers and daily challenges. After securing a nursing position, she was compelled to leave hospital accommodation due to her marriage, confronting discriminatory housing policies in the process. Living in a cramped room with strangers and enduring a hostile environment marked by signs reading “No Blacks, No Irish, No Dogs,” she demonstrated remarkable resilience by giving voice to her lived experiences.

Hennie’s persistence underscores her quest for dignity in a society that sought to deny it. Hennie’s experiences with racism while searching for private housing in the UK highlight the refusal of white landlords to accept black applicants (Holmes, 1988; Rex and Moore, 1967). Many migrants encountered substandard housing and overt discrimination in the post-World War II era (Hampshire, 2005).

Similarly, Caribbean women arriving in the US after the war faced financial difficulties that hindered homeownership, often resulting in their residence in overcrowded project housing. These poor living conditions remain a significant concern for Caribbean women in both the UK and the US (Geronimus, 2023; Mason, 2000; Nazroo, 2003; Sylvester, 2021).

#### Self-efficacy

According to Bandura (1997), self-efficacy refers to an individual’s belief in their capacity to achieve specific goals. For Caribbean women, this sense of confidence is vital for overcoming challenges and adapting to new environments. However, post-migration issues such as trauma and displacement can diminish their self-efficacy, making them more vulnerable to health concerns. Senior Caribbean women often find it difficult to cultivate self-efficacy due to their past experiences as young migrants, complicating their efforts to balance professional careers and motherhood.

Mimi illustrates self-efficacy through her resilience. After facing discrimination in the U.S. that relegated her to a nursing aide role, she made the empowering choice to return to school and ultimately became a qualified nurse, enhancing both her life and that of her children. Here is Mimi’s narrative:

*I was not satisfied working as a nursing assistant; that was never my goal when I left the Caribbean. My goal was to continue my education, so I returned to college and got my nursing qualification. My positive achievement helped my five children pursue a positive educational journey. However, it was tough.*

[Mimi, US, Retired Care Worker, mother, married].

Mimi’s story exemplifies self-efficacy, the belief in one’s capacity to shape their own future despite obstacles. After beginning her career as a nursing assistant, she pursued her ambitions by returning to college to obtain her nursing qualification. This pivotal decision not only transformed her life but also positively influenced her five children, demonstrating how self-efficacy can foster perseverance and inspire future generations.

The Resilience Model, as articulated by Sylvester (2024), explores key aspects of the experiences of senior Caribbean women, including motivation, coping mechanisms, and self-efficacy. These narratives are essential for understanding social resilience in migration studies and underscore the necessity of a critical approach to social injustices. Buckle (2006) notes that resilience is a dynamic concept that evolves as individuals adapt to challenges, as demonstrated by Caribbean women’s experiences (Sylvester, 2021). Nevertheless, it is important to acknowledge that these narratives do not claim to represent the experiences of all Caribbean women regarding migration (Foner, 2009; Peach, 1991).

## Discussion

The article highlights the importance of articulating the narratives of senior Caribbean women through a gendered lens that incorporates Black Feminist Theory and Critical Race Theory. This approach brings their voices to the forefront and focuses on the unique experiences of Caribbean women who migrated to the US and UK following World War II. According to Peach (1968), many first-generation senior Caribbean women made significant contributions to the feminization of migration. Their movement during and after World War II was motivated by various factors, demonstrating their essential roles in the workforce and social economies of both countries. The wave of voluntary migration among Caribbean women is a vital aspect of post-World War II migration theories. Understanding migration decisions requires a gendered approach that examines how social constructs create differences between men and women, as gender shapes migration dynamics (Glick Schiller and Salazar, 2013).

Many first-generation Caribbean women migrated proactively to the UK and the US, seeking employment opportunities rather than merely following their husbands. Many of these unmarried women leveraged migration to achieve financial independence, autonomy, and self-esteem, marking a significant step toward self-empowerment. Some women who initially moved to the UK later relocated to the US in pursuit of better job prospects. This trend illustrates the complexities of voluntary migration among senior Caribbean women transitioning to the US and the UK.

The experiences of Caribbean women differ markedly by socioeconomic status and social class. For instance, those residing in privately owned housing often face different challenges than those living in substandard social housing, who tend to rely more on welfare support. Model (1995) study highlights how income disparities shape the identities of middle-class individuals, in contrast to those of their working-class counterparts, while race remains a critical factor in the challenges they face (Brettell, 2016; Model, 1995; Sylvester, 2021).

This article employs an intersectional framework to explore how race, gender, social class, and power intersect with migration. These dynamics limit opportunities for women and contribute to social injustices, underscoring the need to examine both empowerment and disempowerment in relation to emotional wellbeing and resilience (Crenshaw, 1989; Hill Collins, 1990; Ladson-Billings, 1998; Williams, 2010; Ungar, 2012).

The Caribbean women featured in the study, much like many migrants who arrived after World War II, encountered discrimination that led to low wages and inadequate housing options. As a result, 69% of them resided in substandard private rentals, while only 2% had access to council housing (Peach and Bryon, 1993). Today, these women continue to face significant challenges in securing social housing, including navigating complex welfare systems, limited awareness of their rights, discrimination, and a scarcity of affordable options.

Hennie’s story exemplifies these struggles. Historically, the Caribbean population has been largely exploited by unscrupulous landlords (Peach and Bryon, 1993; Rex and Moore, 1967). Despite these obstacles, many first-generation Caribbean women aspired to homeownership but faced barriers to accessing banking services and obtaining mortgages.

To address these challenges, they established the Pardner Hand Scheme, an informal savings organization managed by women, which allowed them to pool funds for down payments on homes. This initiative has proven effective and remains popular today (Bryan et al., 1985). In one study, 21 out of 22 women demonstrated significant self-efficacy in their pursuit of homeownership, while one woman remained in council housing due to financial constraints (Sylvester, 2021).

Although Caribbean women may be socioeconomically disadvantaged compared to the wider population, evidence suggests that they experience notable upward mobility in housing tenure.

### Resilience and first-generation Caribbean women

This article examines resilience through the lens of first-generation Caribbean women who migrated to the US and the UK after World War II. Their narratives reveal significant diversity from an intersectional viewpoint, often overlooked in discussions of discrimination.

These women encounter societal barriers related to racism, sexism, and class inequality, which underscore their outsider status. However, this awareness can serve as a source of motivation and facilitate the development of effective coping strategies.

Community networks, such as places of worship and volunteer organizations, provide essential support, empowering individuals to tackle these challenges while fostering emotional health and resilience (Alexander, 1996; Reynolds, 2005).

First-generation Caribbean women are often associated with female-headed households, a legacy deeply rooted in Caribbean history. They frequently encounter the “Superwoman” stereotype in both the UK and the US (Beauboeuf-Lafontant, 2003), which imposes expectations that they exhibit qualities such as fearlessness and resilience (Hill Collins, 2000). Consequently, many assume the roles of mothers and breadwinners out of economic necessity, making the “Superwoman” ideal essential for their survival (Mullings and Schulz, 2006).

Beauboeuf-Lafontant (2009) observes that many Black women tend to prioritize the needs of others over their own, particularly Caribbean women who often lack adequate access to welfare and childcare support. This neglect can lead to feelings of disempowerment (Reynolds, 2005). Furthermore, Hooks (1982) argues that Black women adopt a strategy of silence to navigate their circumstances. Despite their considerable resilience, their strengths frequently go unacknowledged. Beauboeuf-Lafontant (2009) stresses that these women have developed various strategies to challenge the “strong Black woman” stereotype.

Nellie’s story poignantly illustrates the challenges she faced while pursuing a work visa in the U. S. As a domestic worker seeking legal citizenship, she encountered racism from her white female employer, underscoring the vulnerabilities of single Caribbean women who migrate for employment opportunities (Hagan, 1994; Kibria et al., 2014; Ladson-Billings, 1998; Sylvester, 2021).

Gee’s story offers a UK perspective on the difficulties faced by a single parent who juggles full-time work and childcare without support from family or state assistance. Hennie’s narrative highlights the emotional distress stemming from the challenges of finding suitable housing, often located in impoverished areas designated for post-war migrants.

Tammi’s experiences illustrate the obstacles Caribbean women face when they are not welcomed in predominantly white-led churches, prompting some to establish their own, Black-led congregations. Currently, nearly two-thirds of churchgoing Christians in the African Caribbean community in the UK and the US attend these Black-led churches rather than traditional Catholic and Protestant denominations (Kalilombe, 1998; Sylvester, 2021).

First-generation Caribbean senior women showcase remarkable resilience as they balance the roles of mother and primary breadwinner. They frequently work long hours for low wages, leading to fatigue and health issues. Additionally, many provide financial support to extended family members in the Caribbean through remittances (Thomas-Hope, 1999).

Senior Caribbean women face considerable challenges, including marginalisation and social isolation that adversely affect their health and wellbeing (Karlsen and Nazroo, 2002a; Karlsen and Nazroo, 2002b). Their responsibilities often hinder their ability to prioritize their own health, complicating their migration experiences. Furthermore, health-related discrimination is frequently overlooked in migration discourse. Geronimus (2023) highlights the detrimental impact of stereotypes on women’s wellbeing, introducing the concept of “weathering.” A nuanced understanding of empowerment and disempowerment is essential, as these factors significantly influence perceptions of health and quality of life (Geronimus, 2023; Wray, 2004).

An intersectional perspective reveals that many first-generation Caribbean women experience feelings of disempowerment and marginalization in both the UK and the US. This framework enhances our comprehension of how race, gender, class, and power intersect to shape their identities and migration experiences. It is crucial to recognize the specific challenges faced by first-generation senior Caribbean women within local Black communities, particularly in the context of events such as the Grenfell Tower fire in 2017 and the Windrush Scandal in 2018. These incidents underscore the troubling treatment of Caribbean Commonwealth individuals by the UK government and highlight the resilience of those who have long been vital members of these communities (Gentleman, 2019; Moore-Bick, 2019).

## Summary

Beginning in the late 1940s, the UK and the US faced significant labor shortages due to World War II and subsequent economic crises. During this time, first-generation Caribbean women emerged as the largest non-white migrant group to these countries, many of whom were in their late teens to early twenties (Brettell, 2016).

These women came from a variety of backgrounds; some held professional qualifications, while others sought better opportunities independently. Today, women represent approximately 40% of the workforce in many Caribbean islands, often serving as the primary earners for their families (Lloyd-Evans and Potter, 2002). Their experiences shed light on the economic factors driving migration and contribute to the broader discourse on the feminization of migration (Castles and Miller, 2004).

Narratives from first-generation Caribbean women reveal challenges related to racism in the UK. Many expressed frustrations over their difficulties finding suitable housing, often forced to live in overcrowded, substandard conditions (Williams, 2010). Analyzing these stories through an intersectional lens uncovers additional barriers in accessing healthcare and childcare, as some African-Caribbean children became entangled with local authorities and the criminal justice system (Williams, 2010; Bryan et al., 1985; Mullings et al., 2006) highlight that these women are frequently overlooked in welfare discussions, resulting in their social and health needs being inadequately addressed.

In the United States, many first-generation Caribbean women face significant challenges when trying to obtain H-1B visas and often encounter discrimination while meeting residency requirements. The principle of family reunification, established in 1965, further complicates their transition to life in the US (Kibria et al., 2014).

An intersectional analysis of first-generation senior Caribbean women reveals that their resilience and empowerment have enabled them to navigate these challenges and establish supportive community networks. Resilience involves confronting emotional losses, economic hardships, and discrimination in an unfamiliar environment. A Critical Race perspective underscores the importance of social equality, emphasizing human agency and power dynamics (Qamar, 2024).

The experiences of these women frequently highlight significant vulnerabilities stemming from their marginalization within UK and US societies (Bryan et al., 1985). Examining their stories through an intersectional lens shows that their complex challenges are shaped by the interplay of race, gender, class, power, age, and migration status (Sowell, 1981; Sylvester, 2021).

## Conclusion

The original research, conducted in 2015, remains relevant today. In the aftermath of World War II, first-generation Caribbean women played a crucial role in shaping the social economies of both the UK and the US. Nevertheless, they faced significant barriers due to their gender, race, social class and migration status. This article underscores the need for further research on how female migrants navigate the challenges of settling into their new environments.

This article has explored the migration experiences of first-generation Caribbean women who travelled to the UK and the US between the 1940s and the 1970s, revealing the resilience they demonstrated in the face of entrenched structural inequalities. Many migrated without the support of extended family networks, yet they adapted to unfamiliar environments, secured employment, raised children, and built new communities of care. Their narratives show how race, gender, class, age, and immigration status are interconnected to shape both their vulnerabilities and their capacities for survival.

Through the lenses of intersectionality, Critical Race Theory, Black Feminist Thought, and the resilience model, their experiences illustrate the multiple pressures they confronted from discriminatory housing markets and limited welfare access to the emotional labor required to navigate everyday racism. Despite these challenges, the women forged meaningful personal and professional lives, often becoming primary earners and community anchors. Their strategies for maintaining cultural identity, fostering belonging, and sustaining wellbeing demonstrate that resilience is not passive endurance but an active, creative process of self-definition (Crenshaw, 1989; Hill Collins, 1990; Ladson-Billings, 1998).

The findings also expose persistent gaps in social policy. Although legislation such as the United States Congress (1964) in the US and the Bleich (2003) in the UK sought to address discrimination, many women continued to face systemic exclusion. The Windrush Scandal underscores the long-term consequences of inadequate immigration protections, while contemporary reforms, including digital ID systems and shifting welfare eligibility, continue to shape migrant women’s access to support. Policy responses in both countries remain fragmented and often fail to recognize the specific needs of female migrants, particularly in relation to childcare, employment recognition, housing, and later-life health (Greve, 2025; Koppe et al., 2025).

The contributions of first-generation Caribbean women to the social and economic fabric of their host societies remain significant yet underacknowledged. Their stories call for more culturally responsive welfare systems, interdisciplinary research, and policy frameworks that reflect the complex realities of migrant women’s lives. Understanding how these women navigated discrimination, built resilience, and sustained their communities offers valuable insights for addressing contemporary migration challenges.

Ultimately, this article contributes a more nuanced account of the emotional and wellbeing dimensions of Caribbean women’s post-war migration and argues for culturally informed research and policy that recognize their diverse identities, strengths, and enduring contributions to the social and economic landscapes of the UK and the US.

As Angelou (1994) reminds us, “we are more than our suffering.” The lives of these Caribbean women embody this truth. Their courage, determination, and insistence on possibility in the face of constraint form an essential chapter in migration history, one that continues to shape present and future generations.

## Data Availability

The datasets presented in this article are not readily available because they are privately stored in a university repository. Requests to access the datasets should be directed to val.sylvester@bcu.ac.uk.
