# Correction: Senior Caribbean women: migration, resilience within the context of intersectionality

**DOI:** 10.3389/fsoc.2026.1832693

**Published:** 2026-04-21

**Authors:** Val Sylvester

**Affiliations:** Birmingham City University, Birmingham, United Kingdom

**Keywords:** communities, Black women, cultural diaspora, culture, first-generation senior Caribbean women, health and wellbeing, intersectionality theory, migrants

The reference for “Sylvester, V. (2004). *The Resilience Model*.” was erroneously written as “Sylvester, V. (2004). *The Resilience Model*.” The Reference List should be cited correctly as “Sylvester, V. (2024). *The Resilience Model*.”

There was a mistake in the caption of Figure 1 as published. “The Resilience Model (Sylvester, 2004).” The Figure 1 caption should be cited correctly as “The Resilience Model (Sylvester, 2024).”

The original version of this article has been updated.

